# Understanding modifiable caregiver factors contributing to child development among young children in rural Malawi

**DOI:** 10.1111/mcn.13698

**Published:** 2024-07-03

**Authors:** Lilia Bliznashka, Odiche Nwabuikwu, Marilyn Ahun, Karoline Becker, Theresa Nnensa, Natalie Roschnik, Monice Kachinjika, Peter Mvula, Alister Munthali, Victoria Ndolo, Mangani Katundu, Kenneth Maleta, Agnes Quisumbing, Melissa Gladstone, Aulo Gelli

**Affiliations:** ^1^ International Food Policy Research Institute Washington District of Columbia USA; ^2^ Global Academy of Agriculture and Food Systems University of Edinburgh Edinburgh Scotland; ^3^ Department of Medicine McGill University Montréal Canada; ^4^ Department of International Development University of Oxford Oxford UK; ^5^ Department of Nutrition and Dietetics Kamuzu University of Health Sciences Blantyre Malawi; ^6^ Save the Children UK London UK; ^7^ Palm Consulting Ltd Zomba Malawi; ^8^ Department of Human Ecology University of Malawi Zomba Malawi; ^9^ Department of Women and Children's Health, Institute of Translational Medicine University of Liverpool Liverpool UK

**Keywords:** child development, dietary diversity, low‐ and middle‐income countries, maternal mental health, stimulation practices, women's empowerment

## Abstract

This study examined modifiable caregiver factors influencing child development in Malawi using baseline data from 1,021 mothers and their children <2 years of age participating in a cluster‐randomized controlled trial implemented in rural Malawi (2022–2025). We fit an evidence‐based theoretical model using structural equation modelling examining four caregiver factors: (1) diet diversity (sum of food groups consumed in the past 24 h), (2) empowerment (assessed using the project‐level Women's Empowerment in Agriculture Index), (3) mental health (assessed using the Self‐Reported Questionnaire, SRQ‐20), and (4) stimulation (number of stimulation activities the mother engaged in the past 3 days). Child development was assessed using the Malawi Development Assessment Tool (norm‐referenced aggregate *Z*‐score). The model controlled for child, caregiver, and household socioeconomic characteristics. Results showed that caregiver dietary diversity was directly associated with higher child development scores (standardized coefficient 0.091 [95% CI 0.027, 0.153]) and lower SRQ‐20 scores −0.058 (−0.111, −0.006). Empowerment was directly associated with higher child development scores (0.071 [0.007, 0.133]), higher stimulation score (0.074 [0.013, 0.140]), higher dietary diversity (0.085 [0.016, 0.145]), and lower SRQ‐20 scores (−0.068 [−0.137, −0.002]). Further, higher empowerment was indirectly associated with improved child development through enhancement of caregiver dietary diversity, with an indirect effect of 0.008 (0.002, 0.018). These findings highlight the important role that caregiver diet and empowerment play in directly influencing child development and other aspects of caregiver well‐being. Interventions aimed at enhancing child development should consider these factors as potential targets to improve outcomes for children and caregivers.

## Key messages

- Caregiver diet and empowerment were associated with child development in children <2 years of age in rural Malawi.
- Caregiver diet was associated with improved caregiver mental health.
- Caregiver empowerment was directly associated with improved caregiver diet, mental health, and stimulation practices.
- Caregiver empowerment was indirectly associated with child development via improved caregiver diet.
- Interventions aimed at enhancing child development can focus on caregiver diet and empowerment as potential targets to improve outcomes for both children and their caregivers.

## Graphical abstract

This study examines modifiable caregiver factors influencing child development in Malawi. Caregiver diet and empowerment were associated with better child development. Both factors were associated with improved caregiver outcomes.

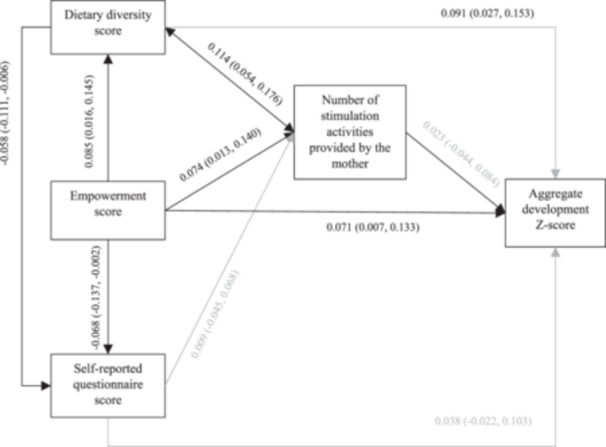

## 1 INTRODUCTION

In low‐ and middle‐income countries (LMICs), 250 million children <5 years of age are at risk of not reaching their developmental potential (Black et al., 2017). In Malawi, it is estimated that 39% of children are at risk of suboptimal child development (Gil et al., 2020; Lu et al., 2020). In 2022, the country's Human Capital Index was 0.41, indicating that children born that year are estimated to be 41% as productive as those who have access to quality education and enjoy the benefits of good health (The World Bank, 2020). This is likely to have adverse effects on later educational attainment and health outcomes (Stein et al., 2023). Strengthening community support for enhancing children's development in these formative years is likely to lead to gains in adult human capital (Campbell et al., 2014; Gertler et al., 2014).

To reach their full developmental potential, children require multiple inputs to thrive, including adequate health, nutrition, opportunities for early learning, safety and security, and responsive care as highlighted by the WHO UNICEF Nurturing Care Framework (Black et al., 2020). Underlying these proximal components is a complex network of enabling environments that encompass caregiver, family, and community processes among other system‐level factors (Black et al., 2020). Although ecological models propose interdependencies among these enabling environments, prior studies in LMICs have predominantly focused on evaluating individual caregiver factors such as maternal mental health (Bennett et al., 2016; Bluett‐Duncan et al., 2021; Burger et al., 2020) or parental stimulation (Frongillo et al., 2017; Jeong et al., 2021), and did not examine the intricate associations between caregiver factors.

In Malawi, little is known about the factors that most influence child development. A handful of studies have assessed the prevalence and severity of suboptimal child development within specific populations. This includes children hospitalised with severe acute malnutrition (van den Heuvel et al., 2017), children attending preschool centres (Murphy et al., 2020), children born preterm (Gladstone et al., 2011), or children exposed to malaria during pregnancy (Weckman et al., 2021). A few studies have examined the effects of nutrition interventions (Phuka et al., 2012; Prado et al., 2020; Prado, Maleta, et al., 2016; Prado, Phuka, et al., 2016), integrated nutrition and parenting interventions (Gelli et al., 2019), or training interventions for early childhood centre staff (Jolley et al., 2022) on child development. However, only one of these studies examined the wider factors influencing child development, focusing on child linear growth, child morbidity, and household socioeconomic characteristics (Phuka et al., 2012). To our knowledge, only three studies have examined caregiver factors contributing to child development in Malawi (Daniel et al., 2021; Lee & Park, 2020; Prado et al., 2017). Using structural equation modelling (SEM) and data from four prospective cohorts, Prado and colleagues examined the influence of child, caregiver and household factors on motor and language development in Burkina Faso, Ghana and Malawi. The home environment, variety of play materials and maternal stimulation were the key caregiver factors which contributed to motor and language development in 18‐month‐old children in Malawi (Prado et al., 2017). More recently, Daniel et al. used SEM to assess the associations between caregiver body mass index, the home environment, child nutritional status and development in children 6–59 months of age discharged from inpatient treatment for severe acute malnutrition. Results showed that caregiver body mass index was associated with child development through its association with the home environment and child nutritional status (Daniel et al., 2021). Lastly, Lee and Park used growth mixture modelling to assess the relationship between maternal perinatal depression trajectories and child development from pregnancy to 2 years of age. Children whose mothers' depression aggravated over time had poorer cognitive and motor development than children whose mothers' depression mitigated over time (Lee & Park, 2020).

Given this limited literature, our objective was to understand the role of modifiable caregiver factors influencing child development in Malawi. Understanding the complex associations between modifiable caregiver factors may help inform the design of interventions aiming to improve child development and unpack the mechanisms through which these interventions work, particularly for those implemented at the community level. Improving intervention design can help better support children and their families living in particularly vulnerable contexts like rural Malawi where the economic situation for families is particularly poor and where families are increasingly subject to extreme weather events like cyclones and droughts (FEWS NET, 2024; IFRC, 2023). In this study, we aimed to deepen and expand knowledge regarding caregiver factors, including those empirically proven in Malawi and others that are theoretically plausible factors but that have not yet been empirically examined in the Malawi context.

## 2 METHODS

### 2.1 Conceptual framework

**Figure 1:**
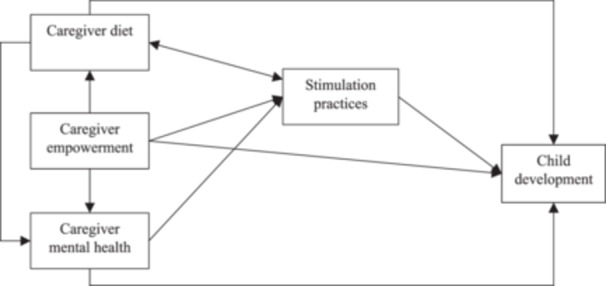
Conceptual model of the associations between caregiver factors and child development. Bidirectional arrows represent correlations.

To examine the associations between caregiver factors and child development, we developed an evidence‐based theoretical model based on the All Children Surviving and Thriving Framework (Black et al., 2020) (Figure 1). We focused on four modifiable caregiver factors where evidence from Malawi is most limited: diet, empowerment, mental health and stimulation practices.

First, evidence from high‐income countries shows that better maternal diet during pregnancy is associated with improved child cognitive, language and socioemotional development (Borge et al., 2017; Saros et al., 2023). Postnatally, maternal diet can influence child development directly through the nutrients provided through breastfeeding and indirectly by affecting women's health, physical activity and caregiving behaviours. However, empirical evidence on the latter is lacking. Regarding caregiver diet and other caregiver factors, macro‐ and micronutrient intake play an important role in the synthesis of neurotransmitters and mood regulators such as serotonin and dopamine (Rechenberg & Humphries, 2013). Lower levels and deficiencies in these nutrients have been linked to perinatal depression (Sparling, Nesbitt, et al., 2017). A healthier diet, generally, and multivitamin supplementation, more specifically, has protective effects against perinatal depression (Sparling, Henschke, et al., 2017). Although most evidence comes from high‐income settings (Sparling, Henschke, et al., 2017; Sparling, Nesbitt, et al., 2017), a handful of studies have demonstrated the protective effects of multivitamin supplementation on perinatal mental health in LMICs (Frith et al., 2009; Smith Fawzi et al., 2007). Yet, evidence is mixed, with studies observing no effect of multivitamin supplementation on perinatal mental health in Indonesia or Malawi (Prado et al., 2012; Stewart et al., 2017). Nevertheless, the physiological role of micronutrients in the synthesis, metabolism, and regulation of neurotransmitters is likely independent of geographical context. Lastly, caregiver diet is likely associated with stimulation. Evidence suggests that more diverse child diets are associated with increased stimulation (Bliznashka, Perumal, et al., 2021; Larson et al., 2017). Given that mothers' and children's diets are highly correlated (Nguyen et al., 2013), it is plausible that more diverse caregiver diets are also associated with increased stimulation. However, empirical support for this association is lacking. Since most evidence on diet diversity and stimulation is cross‐sectional, we model this association as bidirectional since it is unclear whether stimulation influences dietary diversity or vice versa.

Second, with respect to women's empowerment, there is evidence of its direct association with child development (Bliznashka, Udo, et al., 2021; Ewerling et al., 2020). Indirect evidence also shows that women's empowerment is associated with women's diets (Narayanan et al., 2019; Quisumbing et al., 2020), mental health (Hamad et al., 2008; Leight et al., 2022; Scott et al., 2020) and stimulation practices (Bliznashka, Udo, et al., 2021). However, these associations are nuanced, as some empowerment dimensions contribute to improved women's outcomes and others to worsened women's outcomes. For example, ‘asset ownership’ and ‘autonomy in production’ are associated with less diverse diets whereas ‘speaking in public’ is associated with more diverse diets (Quisumbing et al., 2020). Similarly, evidence on the association between women's empowerment and mental health is mixed, with some evidence indicating that more empowered women have better mental health (Leight et al., 2022; Scott et al., 2020) and others suggesting that more empowered women have poorer mental health (Hamad et al., 2008). Still in some contexts, dimensions of women's empowerment are not associated with mental health (Leight et al., 2022). Lastly, emergent evidence indicates that more empowered women, specifically those with higher decision‐making power, provide more stimulation activities to their children (Bliznashka, Udo, et al., 2021). Of note is that both the multidimensional and context‐specific nature of women's empowerment and the diversity of tools used to assess it, make comparisons across studies and cultural settings challenging.

Third, poor post‐partum caregiver mental health is associated with poor child development in LMICs, but relatively little evidence comes from sub‐Saharan Africa (Bluett‐Duncan et al., 2021; Morales et al., 2023). A 2021 systematic review of the associations between perinatal depression and child cognitive development in LMICs did not identify any studies from Malawi (Bluett‐Duncan et al., 2021). Poor mental health has been demonstrated to be associated with poor parenting practices and negative parenting behaviours (Goodman et al., 2020) including in LMICs, though evidence is limited (Huang et al., 2017; Tomlinson et al., 2005).

Fourth, extensive evidence from LMICs suggests that improved stimulation and parenting practices are associated with improved child development outcomes (Frongillo et al., 2017; Jeong et al., 2021).

### 2.2 Participants

We used baseline data from a cluster randomised controlled trial (cRCT) designed to test the independent and combined effects of nutrition‐sensitive social behaviour change with or without unconditional cash transfers on diet adequacy in women of reproductive age and on child development in children <2 years of age (The Maziko Trial Team, 2024). The trial is being conducted in rural Malawi in the districts of Balaka in the Southern Region and Ntcheu in the Central Region. Target areas comprised 156 clusters or communities that included a community‐based childcare centre (CBCC). Women were eligible for inclusion into the trial if they were 15–49 years of age, they self‐reported as pregnant and/or were mothers of children aged <2 years of age, and they lived in the target area. Before trial enrolment, a household census was conducted in target clusters which collected data on household demographics for all household members, including pregnancy status. The census data were used to create a listing of all eligible households. Pregnant women and mothers of children <2 years of age were randomly selected from this household listing for inclusion in the trial. Random selection was stratified by pregnancy status. Between 3 May 2022 and 26 June 2022 the trial enroled 2686 pregnant women or mothers of children <2 years of age living within the CBCCs catchment areas. An average of 18 women were recruited in each cluster, ranging from 7 to 34. Data were collected by trained enumerators using quantitative questionnaires. All enumerators received a 2‐week training before the start of the baseline survey.

### 2.3 Measures

Child gross motor, fine motor, language, and social development were assessed among children aged <2 years at the time of enrolment using the Malawi Development Assessment Tool (MDAT), a culturally relevant tool designed to assess child development in low‐resource African settings (Gladstone et al., 2010). MDAT includes 39–42 items in each of the four domains. Using the MDAT Scoring Application (McCray et al., nd), we calculated MDAT *Z*‐score as an aggregate developmental score and for each of the four domains by comparing raw scores to a reference Malawian population. MDAT was administered to children at home in the presence of their caregivers. Enumerators administering the MDAT received a 3‐day training, including classroom‐based practice, using the standard MDAT administration guidelines. They were checked for at least 90% reliability by a trained nurse with 15 years of experience training on the MDAT. MDAT's reliability and validity have been previously demonstrated (Gladstone et al., 2010). In our sample, internal consistency reliability as measured by Cronbach's *α* was high for all four developmental domains: *α* = 0.95 for gross motor, *α* = 0.94 for fine motor, *α* = 0.90 for language, and *α* = 0.95 for social. We used the aggregate MDAT *Z*‐score in the main analysis. As a sensitivity analysis, we refit the conceptual model separately for each domain.

Caregiver diet was assessed using a quantitative multi‐pass 24‐h recall (Gibson & Ferguson, 2008). For simplicity, we calculated women's dietary diversity score (range 0–10) by grouping foods into 10 food groups: (1) grains, white roots and tubers and plantains; (2) pulses; (3) nuts and seeds; (4) dairy; (5) meat, seafood, and poultry; (6) eggs; (7) dark‐green leafy vegetables; (8) vitamin A–rich fruits and vegetables; (9) other vegetables; and (10) other fruits. A score of five or above was used to define minimum dietary diversity for women (MDD‐W) (FAO, 2021).

Women's empowerment was assessed using the project‐level Women's Empowerment in Agriculture Index (pro‐WEAI) (Malapit et al., 2019). Pro‐WEAI consists of 12 equally weighted indicators measuring three domains of women's empowerment: intrinsic agency (autonomy in income, self‐efficacy, attitudes about intimate partner violence and respect among household members), instrumental agency (input in productive decisions, land and other asset ownership, access to and decisions on financial services, control over use of income, work balance and visiting important locations) and collective agency (group membership and influential group membership) (Malapit et al., 2019). The WEAI has been previously validated among rural populations in Malawi (Connors et al., 2023; Malapit et al., 2014; Mponela et al., 2021; Onah et al., 2021). Since we hypothesised that all three domains (and the indicators comprising them) were relevant for the caregiver factors included in our model, we used the aggregate pro‐WEAI (range 0–1) in our analyses, with higher numbers indicating greater empowerment. Empowerment adequacy scores for each indicator are shown in Supporting Information S1: Table 1. To further explore the drivers of change and because not all empowerment domains are relevant for child development (Bliznashka, Udo, et al., 2021), we performed additional exploratory analyses by refitting the conceptual model separately for each individual women's empowerment indicator.

Caregiver mental health was assessed using the World Health Organization (WHO) Self‐Reported Questionnaire (SRQ‐20) (World Health Organization, 1994). The SRQ‐20 consist of 20 questions (yes/no answer) exploring symptoms of depression, anxiety and somatic manifestation of distress. All questions were asked in a private area, out of sight or earshot from household or community members. The SRQ‐20 has been previously validated in Malawi for diagnosis of symptoms consistent with major/minor depressive disorder against a clinical diagnosis using Diagnostic and Statistical Manual of Mental Disorders (DSM)‐IV criteria (Stewart et al., 2009). Higher SRQ‐20 scores indicate worse mental health. A score of 8 or above was used to define symptoms consistent with major/minor depression (Stewart et al., 2009). There was high internal consistency among SRQ‐20 items (*α* = 0.84) in our sample. The proportions of women with each SRQ‐20 symptom are shown in Supporting Information S1: Table 2.

Caregiver practices were assessed using one section from the Family Care Indicators (Kariger et al., 2012). Caregivers reported on the number of times they engaged in 14 types of stimulation activities with their child in the 3 days preceding the interview. This version was previously translated, back translated, adapted, piloted and re‐adapted in Malawi (Prado et al., 2016). It has since been used in other studies in Malawi with good sensitivity to change (Weckman et al., 2021). We constructed binary indicators for whether caregivers engaged in each activity at least once and summed them up into a total stimulation score (range 0–14). The proportions of mothers engaging in each activity are shown in Supporting Information S1: Table 3.

### 2.4 Statistical analysis

A total of 1381 caregiver‐child pairs were assessed at baseline. The remaining 1305 women enroled in the trial reported being pregnant at baseline. All caregivers were the biological mothers of the children. We restricted the analytic sample to mother–child pairs with available data on child development and the four caregiver factors of interest (diet, empowerment, mental health and stimulation practices). Women with incomplete data on any of the 12 empowerment indicators were excluded, in line with pro‐WEAI construction guidelines (Seymour et al., 2023). The final analytic sample consisted of 1021 mother–child pairs (Supporting Information S1: Figure 1). Differences between included and excluded pairs were assessed using a Wald test, considered significant at *p* < 0.05.

SEM was used to fit our conceptual model (Kline, 2015). Direct, indirect and total effects were estimated, where indirect effects were the product of two direct effects and total effects were the sum of direct and indirect effects. The term ‘effects’ was used noncausally given the cross‐sectional nature of the data, in line with SEM nomenclature (Kline, 2015). Standard errors were clustered at the cluster level to account for the multi‐level nature of the data. Randomisation of clusters into the trial intervention arms was conducted after baseline data were collected (The Maziko Trial Team, 2024), and we therefore did not control for intervention arm assignment in our analyses. All direct paths controlled for the following sociodemographic characteristics to minimise potential confounding: child sex; caregiver age, education (none vs. primary or higher), and marital status (married/cohabitating vs. single/divorced/separated/widowed); household size and expenditures. Direct paths to child stimulation, caregiver diet and caregiver mental health also controlled for child age. The direct paths to MDAT did not control for child age since the aggregate and domain‐specific *Z*‐scores we used were age‐standardized. To account for prior, cumulative early life exposures and given that child height may influence the stimulation and engagement children receive (Brown & Pollitt, 1996), the direct paths to MDAT and stimulation also controlled for child length‐for‐age *Z*‐score, calculated using the 2006 WHO Child Growth Standards (World Health Organization, 2006). Missing data on covariates (<5%) were imputed using cluster‐level means. Absolute model fit was determined acceptable based on the following criteria: Comparative Fit Index (CFI) ≥ 0.90, root mean square error of approximation (RMSEA) ≤ 0.08, and standardized root mean squared residual (SRMR) ≤ 0.08 (Hu & Bentler, 1999). With 59 free parameters to estimate, we had a ratio of 17 observations per parameter, meeting the minimum requirement of 10 observations per parameter often used in SEM (Kline, 2015). We calculated 95% bias‐corrected bootstrapped confidence intervals (referred to as 95% CI for brevity) with 5000 draws to test the significance of the direct, indirect and total effects (Preacher & Hayes, 2008).

Subgroup analyses were conducted to explore if the model parameters differed by child age (<6 months vs. 6–24 months of age), caregiver education and marital status. To test for invariance between groups, a Wald test was conducted for difference between a model where all parameters were constrained to be equal across groups and a model where all parameters were unconstrained across groups. Descriptive statistics were prepared in Stata 17 (StataCorp, 2021) and model fitting and subgroup analyses were conducted in MPlus version 8.4 (Muthén & Muthén, 2017).

### 2.5 Ethical statement

Ethical approval for the trial was obtained from the institutional review boards of the University of Malawi (protocol number: P. 02/22/128) and the International Food Policy Research Institute (protocol number: 7490). Verbal information on the study was provided and written informed consent was obtained from adult women or the parents/guardians of women <18 years of age.

## 3 RESULTS

### 3.1 Sample descriptives

Children in our sample (*n* = 1021) were 9.8 months of age on average and mean aggregate MDAT *Z*‐scores were 0.7 (SD 1.1) (Table 1). Caregivers were 25 years old on average and nearly two‐thirds had not completed any formal education. Caregiver diet was poor, with 13% of women meeting minimum dietary diversity. One‐fifth of caregivers had symptoms consistent with depression. Caregivers on average engaged in two stimulation activities (out of 14) in the 3 days before the survey.

**Table 1:** Household, caregiver and child characteristics of the 1021 mother–child pairs in the analytic sample.

	Mean ± SD or *N* (%)
*N*	1021
*Child characteristics*	
Child is a boy	532 (52.1%)
Age (in months)	9.8 ± 7.4
Aggregate development *Z*‐score	0.7 ± 1.1
Gross motor development *Z*‐score	0.7 ± 1.3
Fine motor development *Z*‐score	0.7 ± 1.3
Language development *Z*‐score	0.2 ± 1.2
Social development *Z*‐score	0.6 ± 1.0
Length‐for‐age *Z*‐score (LAZ)	−1.4 ± 1.4
*Caregiver characteristics*	
Age (in years)	25.2 ± 6.8
No formal education completed	644 (63.1%)
Married or cohabitating	864 (84.6%)
Dietary diversity score (0–10)	3.3 ± 1.0
Met minimum dietary diversity (dietary diversity score ≥5)	132 (12.9%)
Empowerment score (0‐1)	0.6 ± 0.2
Considered empowered (adequate in ≥8 out of the 10 empowerment indicators)	27.9%
Self‐reported questionnaire score (SRQ, 0–20)	4.3 ± 4.0
Has symptoms consistent with major/minor depression (SRQ ≥ 8)	213 (20.9%)
Number of stimulation activities provided by the mother in the past 3 days (0–14)	2.1 ± 1.7
*Household characteristics*	
Size	4.8 ± 1.8
Per capita monthly expenditures (MWK)^a^	8335 ± 8144

^a^ MWK, Malawian Kwacha. $1 USD was approximately 1700 MKW in June 2024.

Overall, children included in the analysis were similar to those who were excluded (*n* = 360) due to incomplete data on child development or the caregiver factors of interest (Supporting Information S1: Table 4). Included and excluded caregivers were similar in terms of diet, empowerment and stimulation practices. However, excluded caregivers had slightly higher SRQ‐20 scores and were more likely to have not completed any formal education. In addition, excluded caregivers were substantially less likely to be married/cohabitating than included caregivers. Excluded caregivers also came from slightly smaller households (Supporting Information S1: Table 4). Most women were excluded from the analysis due to missing data on empowerment (*N* = 330, 92%).

### 3.2 Structural equation model

**Figure 2:**
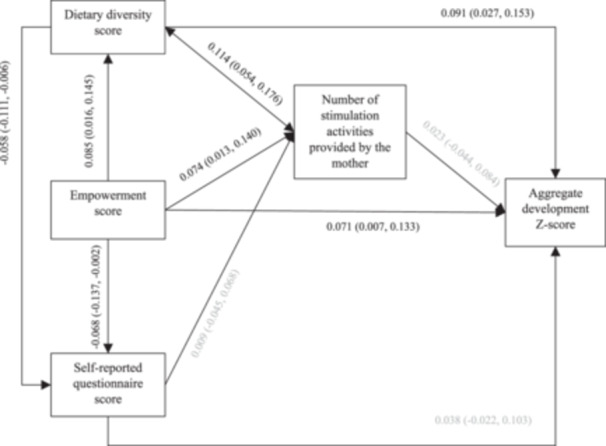
Standardized direct effects and bias‐corrected bootstrapped 95% confidence intervals. Grey paths represent estimates not significant at the 5% level. Estimates controlled for child age and sex, maternal age, education, and marital status, household size and expenditures and district. The direct paths to child development, which was age‐standardized, did not control for child age. The direct paths to child development and stimulation practices also controlled for length‐for‐age *Z*‐score.

Our model showed adequate fit: CFI = 0.942, RMSEA = 0.059 and SRMR = 0.019. After controlling for confounding, higher dietary diversity was directly associated with higher child development scores (standardized coefficient 0.091 [95% CI 0.027, 0.153]), lower SRQ‐20 scores (−0.058 [95% CI −0.111, −0.006]), and higher stimulation score (0.114 [95% CI 0.054, 0.176]) (Figure 2). Empowerment was directly associated with higher child development scores (0.071 [95% CI 0.007, 0.133]), higher dietary diversity (0.085 [95% CI 0.016, 0.145]), lower SRQ‐20 (−0.068 [95% CI −0.137, −0.002]) and higher stimulation scores (0.074 [95% CI 0.014, 0.140]). In addition, higher empowerment was indirectly associated with higher child development scores through improved dietary diversity, but the strength of this association was small (Table 2). No other indirect associations were found. Unstandardised direct and indirect effects are shown in Supporting Information S1: Figure 2 and Table 5, respectively.

**Table 2:** Standardized indirect effects on child development through caregiver characteristics.

	Standardized coefficient (bias‐corrected bootstrapped 95% CI)
Dietary diversity score → self‐reported questionnaire score → aggregate development *Z*‐score	−0.002 (−0.009, 0.000)
Dietary diversity score → self‐reported questionnaire score → stimulation → aggregate development *Z*‐score	0.000 (0.000, 0.000)
Empowerment → stimulation → aggregate development *Z*‐score	0.002 (−0.002, 0.009)
Empowerment → dietary diversity score → aggregate development *Z*‐score	0.008 (0.002, 0.018)
Empowerment → self‐reported questionnaire score → aggregate development *Z*‐score	−0.003 (−0.011, 0.001)
Empowerment → self‐reported questionnaire score → stimulation → aggregate development *Z*‐score	0.000 (0.000, 0.000)
Empowerment → dietary diversity score → self‐reported questionnaire score → aggregate development *Z*‐score	0.000 (0.000, 0.000)
Empowerment → dietary diversity score → self‐reported questionnaire score → stimulation → aggregate development *Z*‐score	0.000 (0.000, 0.000)
Self‐reported questionnaire score → stimulation → aggregate development Z‐score	0.000 (−0.001, 0.005)

In sensitivity analyses where we refit the model separately for each developmental domain, higher dietary diversity was associated with higher gross motor scores (Supporting Information S1: Figure 3), higher SRQ‐20 scores were associated with higher fine motor scores (Supporting Information S1: Figure 4), and higher empowerment was associated with higher language scores (Supporting Information S1: Figure 5). Further, higher dietary diversity, higher empowerment and higher stimulation scores contributed to higher social development scores (Supporting Information S1: Figure 6). Consistent with the findings for aggregate development scores, higher empowerment was indirectly associated with higher gross motor and social development scores through improved dietary diversity (Supporting Information S1: Table 6). In addition, higher empowerment and higher dietary diversity were indirectly associated with lower fine motor scores through SRQ‐20 scores, whereas higher empowerment was indirectly associated with higher social development scores through higher stimulation scores (Supporting Information S1: Table 5).

### 3.3 Exploratory analyses by women's empowerment domain

Higher empowerment in terms of ‘work balance’, ‘visiting important locations’, ‘respect among household members’, and ‘self‐efficacy’ was directly associated with higher child development scores (Supporting Information S1: Table 7), with the magnitude of all these associations larger than for total empowerment, and ranging from 0.071 (95% CI 0.012, 0.126) for ‘work balance’ to 0.110 (95% CI 0.044, 0.172) for ‘respect among household members’. Higher ‘input in productive decisions’, ‘access to and decisions on financial services’, and ‘self‐efficacy’ were associated with higher dietary diversity score, lower SRQ‐20 scores, and higher stimulation scores. In addition, ‘land and other asset ownership’ and ‘control over income use’ were associated with improved dietary diversity and stimulation scores. Higher empowerment in terms of ‘group membership’, ‘influential group membership’, and ‘work balance’ was associated with higher stimulation scores. Lastly, higher ‘group membership’ was associated with better dietary diversity and ‘higher respect among household members’ with lower SRQ‐20 scores (Supporting Information S1: Table 6).

The positive indirect effect between empowerment and child development scores via dietary diversity remained significant in the models for 7 of the 12 empowerment indicators with the largest indirect effect for ‘land and other asset ownership’: 0.014 (95% CI 0.04, 0.028) (Supporting Information S1: Table 8).

### 3.4 Subgroup analyses

First, for child age, we compared a constrained model with all parameters equal between children <6 months of age and children 6–24 months of age with an unconstrained model where all parameters were freely estimated across the two groups. The unconstrained model was not a significantly better fit (*p* = 0.539). In children <6 months of age, higher dietary diversity was directly associated with higher child development scores and higher empowerment was indirectly associated with better child development through higher dietary diversity (Supporting Information S1: Table 9). In contrast, in children 6–24 months of age, higher stimulation scores were directly associated, and higher empowerment was both directly and indirectly (via stimulation) associated, with higher child development scores.

Second, we compared a constrained model with all parameters equal between caregivers without completed formal education and caregivers with primary or higher education with an unconstrained model where all parameters were freely estimated across the two groups. The latter model was a significantly better fit (*p* = 0.037). None of the four caregiver factors were directly associated with child development in either group (Supporting Information S1: Table 8). Indirectly, higher empowerment was associated with improved child development through improved dietary diversity.

Third, compared with the constrained model where all parameters were equal between married/cohabitating caregivers and unmarried caregivers, the unconstrained model where all parameters were freely estimated across the two groups was not a significantly better fit (*p* = 0.294). We observed that higher dietary diversity was directly associated, and higher empowerment scores were both directly and indirectly (via dietary diversity) associated, with improved child development among married/cohabitating caregivers (Supporting Information S1: Table 8).

## 4 DISCUSSION

In this study, we examined how four modifiable caregiver factors— diet, empowerment, mental health and stimulation practices—were associated with development in children <2 years of age in rural Malawi. We built on prior literature from Malawi, which has largely examined individual caregiver factors—mental health and stimulation (Lee & Park, 2020; Prado et al., 2017), and other LMICs by simultaneously examining four factors and the complex inter‐relationships that exist between them. Our findings showed that caregiver diet was directly associated with improved child development and caregiver outcomes, whereas associations between empowerment and child development were both direct and indirect. However, associations with women's empowerment were highly nuanced, with different empowerment domains associated with different child and/or caregiver outcomes. Caregiver mental health and stimulation practices were associated with fine motor and social development, respectively, but not with overall child development.

Our finding, which establishes a direct and positive association between post‐natal caregiver diet and post‐natal child development, builds upon previous research conducted in high‐income countries that primarily focused on the connection between prenatal caregiver diet and child development (Borge et al., 2017; Saros et al., 2023). The observation that the association between caregiver diet and child development was more pronounced and statistically significant among children <6 months of age, but not children 6–24 months of age, suggests that caregiver diet may have exerted a direct influence on child development though breastfeeding. Nonetheless, it is essential to interpret these findings cautiously as for these subgroups the unconstrained model did not significantly outperform the constrained model and our study was not explicitly powered for subgroup analysis or designed to explore the precise mechanisms through which caregiver diet impacted child development. Moreover, an enhanced caregiver diet may also serve as an indicator or proxy for the overall physical and financial well‐being of caregivers, rather than solely indicating improved nutrient intake. Consequently, disentangling the direct and indirect mechanisms through which caregiver diet may have impacted child development is a complex endeavour. Future research should consider explicit designs to assess these pathways linking caregiver diet and child development in the post‐natal period.

With respect to caregiver empowerment, consistent with prior literature we found that higher empowerment was associated with improved child development and caregiver outcomes (Bliznashka, Udo, et al., 2021; Ewerling et al., 2020; Leight et al., 2022; Quisumbing et al., 2020). Further, different empowerment domains contributed to different child and/or caregiver outcomes. For example, total empowerment was associated with overall, language, and social development, but not with fine or gross motor development. Empowerment in terms of ‘work balance’, ‘visiting important locations’, ‘respect among household members’, and ‘self‐efficacy’ was associated with improved child development and caregiver outcomes, whereas empowerment in terms of ‘land and asset ownership’, ‘group membership’, and ‘input into productive decisions’ was only associated with improved caregiver outcomes. These results point towards the need to improve female caregivers' work balance, mobility, intrahousehold relations and self‐efficacy to improve both child and caregiver outcomes. The findings also suggest that interventions seeking to improve both child development and caregiver outcomes may want to focus more on the former set of domains (i.e., ‘work balance’, ‘visiting important locations’, ‘respect among household members’, and ‘self‐efficacy’). The importance of empowerment in terms of ‘work balance’ and ‘respect among household members’ highlights the broader role of the family as a unit, and the complex role played by caregiving. A recent systematic review of father‐inclusive interventions demonstrated benefits for multiple individuals within the family beyond the mother and the child (Jeong et al., 2023). Together these findings indicate that future interventions to improve child and caregiver outcomes in rural Malawi should seek to engage other family members both in caregiving and as champions of women's empowerment.

Further, we found that caregiver empowerment was indirectly associated with overall child development through improved caregiver diet, expanding existing literature on the hypothesized pathways through which women's empowerment may influence child development (Bliznashka, Udo, et al., 2021). In line with this prior literature, we found that increased stimulation was a pathway through which empowerment improved social development in children <2 years of age and overall development among children 6–24 months of age. Caution is warranted in interpreting findings for children 6–24 months of age as our study was not a priori powered for subgroup analysis and the unconstrainted model was not significantly better than the constrained model.

Caregiver mental health was not associated with overall, gross motor, language or social development either directly or indirectly. These findings build on the limited literature on the topic from sub‐Saharan Africa (Bluett‐Duncan et al., 2021) and Malawi in particular where one study has demonstrated a relationship between maternal perinatal depression and poorer cognitive and motor development in children <2 years of age (Lee & Park, 2020). This lack of associations could be explained by the sociocultural context: caregivers in our sample came from larger households (five members on average) and the majority were empowered in terms of ‘respect among household members’ and ‘self‐efficacy’, indicating the presence of social support, albeit by proxy, which may have helped alleviate any negative effects of poor caregiver mental health on child development. Other differences in study design, such as the use of different mental health assessment tools and measurements at single versus multiple time points, can help explain the difference in findings between our study and others that have observed an association (Lee & Park, 2020). However, we also observed that mental health was inversely associated with fine motor development such that women with higher SRQ‐20 scores (i.e., worse mental health) had children with better fine motor scores. Given the extensive theoretical and empirical literature on the negative effects of maternal depression on child development (Bluett‐Duncan et al., 2021; Gelaye et al., 2016; Morales et al., 2023), it is likely that this association was spurious given the large number of associations we tested.

Lastly, we found that stimulation practices were associated with overall child development among children 6–24 months of age and with social development in the full sample. Previously in Malawi, Prado and colleagues observed an association between maternal stimulation and child motor and language development in 18‐month‐old children (Prado et al., 2017). The lack of association in the younger age group or with motor and language development in our sample may be due to the different assessment tools, lack of sensitivity in this age group in the measures we used, or lack of variability given that some stimulation activities were not relevant for this age group. Further our study was not explicitly designed for subgroup analyses and may have been underpowered to detect differences in specific age groups. Future studies may consider using more comprehensive tools that capture more caregiving and parenting aspects such as the Home Observation for Measurement of the Environment (HOME) Inventory. The advantages of such tools should be carefully weighed against their disadvantages in large field settings including the longer time to administer and more substantive training required.

With respect to associations between caregiver factors, one finding worth highlighting is the association between more diverse caregiver diets and more diverse stimulation. Limited prior literature has demonstrated this association in children 12–18 months of age and 36–59 months of age (Bliznashka, Perumal, et al., 2021; Larson et al., 2017). We built on this literature by demonstrating this association in children <2 years of age. However, given the cross‐sectional nature of the data, we cannot determine the directionality of this association. Nevertheless, these findings may indicate that women may be able to translate diversity from one facet to another, a speculation supported by the translation hypothesis that caregivers are able to translate responsiveness from one facet to another (Landry et al., 2006), which has at least partial empirical support from India and Pakistan (Bliznashka et al., 2022; Nahar et al., 2009). Despite this emerging evidence that social behaviour change communication and parenting interventions may have cross‐over effects on caregiver responsiveness (Bliznashka et al., 2022), additional research is needed to formally test this translation hypothesis and quantify cross‐over effects in different settings as well as to fully unpack the association between caregiver diet diversity and stimulation observed here and in prior literature (Bliznashka, Perumal, et al., 2021; Larson et al., 2017).

Our study had several strengths including developing an a priori conceptual model grounded in theoretical and empirical literature before model testing, lack of statistical model modifications, large sample size and exploratory subgroup analyses. Nevertheless, the study findings should be interpreted with caution for several reasons. First, our analyses were cross‐sectional and none of the results highlighted here are causal. Future research should examine the associations between caregiver factors and child development longitudinally to establish temporal precedence, causality and to assess how these associations evolve over time. Second, we assessed multiple caregiver factors, associations and hypotheses and our analyses are subject to multiple hypothesis testing. Given the limited literature from Malawi, our analyses were exploratory and hypotheses generating in nature. We therefore did not adjust for multiple comparisons. Third, women excluded due to missing data were substantially less likely to be married/cohabitating compared to women included in our analyses. As a result, our findings might not generalise to unmarried women. Other differences in mental health scores and education level observed between included and excluded women were small in magnitude but may further limit generalisability. Fourth, all caregiver factors relied on self‐reported measures and are thus subject to social desirability and recall bias. To mitigate these biases, the diet, stimulation and child development assessments were administered by a different enumerator on the day following the mental health and empowerment assessments. Further, because intervention arm assignment was conducted after baseline data collection, it is unlikely that knowledge of receipt of programme intervention or expectation of specific benefits biased reporting since women were not aware of the planned interventions or their benefits. Finally, although the model estimation controlled for several confounders, unmeasured or unobserved confounders may have influenced the results. Future studies may test the robustness of our findings by including a wider range of factors from the All Children Surviving and Thriving Framework (Black et al., 2020) and other potential confounders.

Despite these limitations, our findings serve to inform the design of future interventions and programmes to improve child development and caregiver outcomes in rural Malawi. The Government of Malawi already recognises the need for multi‐sectoral approaches to improve child development and nutrition (Government of Malawi, 2019) and the importance of improving caregiver wellbeing and gender equality through male engagement (Ministry of Gender Community Development & Social Welfare, 2023). This research reinforces the need to focus on caregivers and to implement interventions that combine child‐focused packages with caregiver‐focused packages like ‘Caring for the Caregiver’ which focuses on caregiver health and emotional well‐being. Testing the effectiveness of such combined interventions is underway in Malawi (The Maziko Trial Team, 2024). Our results further indicated that work balance, mobility, intrahousehold relations, and self‐efficacy are key aspects of caregiver's empowerment and well‐being to improve in the rural Malawi context. Although we found no relationship between maternal mental health and child development, future programmes should still consider improving maternal mental health not only as a way to improve child and caregiver outcomes (Bluett‐Duncan et al., 2021; Gelaye et al., 2016; Morales et al., 2023), but also as an important health outcome in‐and‐of‐itself (United Nations, 2015).

## 5 CONCLUSION

In conclusion, this study examined four modifiable caregiver factors (diet, empowerment, mental health and stimulation practices) and their influence on development in children <2 years of age in rural Malawi. Results showed that caregiver diet and empowerment were the most prominent factors associated with improved child development and caregiver outcomes. Interventions seeking to improve both caregiver diets and empowerment may have synergistic or additive effects. Future research should examine the longitudinal associations between caregiver factors and child development as well as quantify indirect intervention effects on child development through caregiver diet and empowerment.

## AUTHOR CONTRIBUTIONS

Natalie Roschnik, Monice Kachinjika, Mangani Katundu, Agnes Quisumbing and Aulo Gelli designed the study trial. Lilia Bliznashka, Marilyn Ahun, Natalie Roschnik, Monice Kachinjika, Kenneth Maleta, Agnes Quisumbing, Melissa Gladstone and Aulo Gelli wrote the trial protocol. Lilia Bliznashka and Aulo Gelli conceptualized the analyses presented here. Theresa Nnensa, Mangani Katundu, Peter Mvula, Alister Munthali, Victoria Ndolo and Kenneth Maleta implemented the baseline survey and supervised data collection. Odiche Nwabuikwu and Karoline Becker performed data cleaning and curation. Lilia Bliznashka analyzed the data and drafted the manuscript. All authors interpreted the results, and reviewed and approved the final version of the manuscript. Lilia Bliznashka had final responsibility for submitting the manuscript for publication.

## CONFLICT OF INTEREST STATEMENT

The authors declare no conflict of interest.

## Supporting information

Supporting information.

## Data Availability

The data that support the findings of this study are available from the corresponding author upon reasonable request.
