# Supplementary material for: Understanding modifiable caregiver factors contributing to child development among young children in rural Malawi
Source: Matern Child Nutr. 2024 Jul 3;20(4):e13698. doi: 10.1111/mcn.13698 (PMC11574655; doi:10.1111/mcn.13698)
Supplement: Supplementary file 1 — Supporting information. [file MCN-20-e13698-s001.docx]

**Supplemental Table 1** Mean empowerment scores and proportion adequacy for each of the 12 indicators comprising project-level Women’s Empowerment in Agriculture Index (pro-WEAI)

|  | **Mean ± SD**  **or N (%)** |
| --- | --- |
| N | 1021 |
| Input in productive decisions (range 0-8) | 2.1±1.1 |
| Ownership of land and other assets (range 0-14) | 4.3±1.7 |
| Access to and decisions on financial services (range 0-6) | 0.8±0.8 |
| Control over use of income (range 0-8) | 1.6±1.1 |
| Autonomy in income (range -9 to 9) | 0.8±1.7 |
| Group membership (range 0-9) | 0.7±0.9 |
| Membership in influential groups (range 0-9) | 0.6±0.9 |
| Work balance (range 0-24) | 11.8±3.7 |
| Visiting important locations (range 0-6) | 1.6±1.1 |
| Respect among household members (range 0-4) | 3.3±1.0 |
| Attitudes about intimate partner violence against women (range 0-5) | 4.7±0.8 |
| Self-efficacy (range 4-20) | 15.7±4.1 |
| Adequacy in input in productive decisions (respondent makes decisions solely, has input into joint decision-making, or feels that they can make decisions if they want to for all agricultural activities their household performs) | 923 (90.4%) |
| Adequacy in ownership of land and other assets (respondent owns solely or jointly three assets or land) | 926 (90.7%) |
| Adequacy in access to and decisions on financial services (respondent’s household used a source of credit in the past year and the respondent made at least one sole or joint decision about it; or respondent’s household did not use any credit source in the past year but could have if wanted to from at least one source; or respondent has sole or joint access to a financial account) | 572 (56.0%) |
| Adequacy in control over use of income (respondent participates in decisions on income and output from all agricultural and non-agricultural activities) | 609 (59.6%) |
| Adequacy in autonomy in income (relative autonomy index ≥1) | 476 (46.6%) |
| Adequacy in group membership (respondent is active in ≥1 group) | 491 (48.1%) |
| Adequacy in membership in influential groups (respondent is active in ≥1 influential group) | 389 (38.1%) |
| Adequacy in work balance (respondent works <10.5 hours daily) | 356 (34.9%) |
| Adequacy in visiting important locations (respondent visits city, market, or relative once per week; or visits health facility or public meeting once per month) | 632 (61.9%) |
| Adequacy in respect among household members (all conditions met with respect to at least one other adult household member) | 607 (59.5%) |
| Adequacy in attitudes about intimate partner violence against women (believes that a man household member is not justified hitting a woman in all five suggested scenarios) | 838 (82.1%) |
| Adequacy in self-efficacy (self-efficacy score ≥16) | 617 (60.4%) |

**Supplemental Table 2** Proportion of caregivers reporting experiencing each symptom on the Self-Reported Questionnaire (SRQ)

|  | **N (%)** |
| --- | --- |
| N | 1021 |
| Often has headaches | 540 (52.9%) |
| Poor appetite | 155 (15.2%) |
| Poor sleep | 278 (27.2%) |
| Shaking hands | 121 (11.9%) |
| Feeling nervous, tense or worried | 392 (38.4%) |
| Easily frightened | 158 (15.5%) |
| Poor digestion | 61 (6.0%) |
| Has trouble thinking clearly | 225 (22.0%) |
| Feeling unhappy | 298 (29.2%) |
| Cries more than usual | 51 (5.0%) |
| Hard to enjoy daily activities | 164 (16.1%) |
| Hard to make decisions | 174 (17%) |
| Daily work is suffering | 201 (19.7%) |
| Cannot play a useful part in life | 241 (23.6%) |
| Lost interest in things | 196 (19.2%) |
| Feeling worthless | 212 (20.8%) |
| Thoughts of ending one's life | 56 (5.5%) |
| Feeling tired all the time | 274 (26.8%) |
| Uncomfortable feelings in one's stomach | 332 (32.5%) |
| Easily tired | 281 (27.5%) |

**Supplemental Table 3** Proportion of caregivers reporting engaging in each stimulation activity in the 3 days preceding the survey

|  | **N (%)** |
| --- | --- |
| N | 1021 |
| Read books or looked at picture books | 49 (4.8%) |
| Told stories | 24 (2.4%) |
| Sang songs including lullabies | 389 (38.1%) |
| Chatted with child while doing chores or other task | 648 (63.5%) |
| Took child outside the home, compound, yard or enclosure | 518 (50.7%) |
| Played at physical activities with child (dancing, jumping, running, etc.) | 331 (32.4%) |
| Helped child learn letters or numbers | 25 (2.4%) |
| Helped learn shapes or colors | 9 (0.9%) |
| Drew objects in sand or with pen and paper for child | 47 (4.6%) |
| Constructed objects (from paper, wire, mud, etc.) with child | 35 (3.4%) |
| Identified plants, animals, or the natural environment for child | 72 (7.1%) |
| Taught English words | 23 (2.3%) |
| Taught child the names and uses of new object | 15 (1.5%) |
| Played with child in some other way not described here | 1 (0.1%) |

**Supplemental Table 4** Descriptive characteristics of the caregivers and children included in the analytic sample and those excluded due to missing data on caregiver factors and/or child development

|  | **Included in the analysis** | **Excluded from the analysis** |  |
| --- | --- | --- | --- |
|  | **Mean ± SD or N (%)** | **Mean ± SD or N (%)** | **p-value for differences** |
| N | 1021 | 360 |  |
| *Child characteristics* |  |  |  |
| Child is a boy | 532 (52.1%) | 185 (51.3%) | 0.795 |
| Age (in months) | 9.8±7.4 | 10.1±7.3 | 0.530 |
| Aggregate development Z-score | 0.7±1.1 | 0.5±1.8 | 0.058 |
| Length-for-age Z-score | -1.4±1.4 | -1.5±1.2 | 0.495 |
|  |  |  |  |
| *Caregiver characteristics* |  |  |  |
| Age (in years) | 25.4±7.1 | 25.2±7.1 | 0.716 |
| No formal education completed | 642 (62.9%) | 251 (69.7%) | 0.028 |
| Married or cohabitating | 859 (84.1%) | 153 (42.4%) | <0.001 |
| Dietary diversity score (0-10) | 3.3±1.0 | 3.4±1.0 | 0.189 |
| Empowerment score | 0.6±0.2 | 0.6±0.1 | 0.852 |
| Self-reported questionnaire score (0-20) | 4.3±4.0 | 4.9±4.5 | 0.042 |
| Number of stimulation activities provided by the mother in the past 3 days (0-14) | 2.1±1.7 | 2.0±1.5 | 0.342 |
|  |  |  |  |
| *Household characteristics* |  |  |  |
| Size | 4.8±1.8 | 4.4±1.9 | 0.005 |
| Per capita monthly expenditures (MWK)^1^ | 8,3418±8,155 | 8,023±12,019 | 0.655 |

^1^ MWK, Malawian Kwacha. $1 USD is approximately 1700 MKW.

**Supplemental Table 5** Unstandardized indirect effects on child development through caregiver characteristics

|  | **Unstandardized coefficient**  **(Bias-corrected bootstrapped 95% CI)** |
| --- | --- |
| Dietary diversity score → Self-reported questionnaire score → Aggregate development Z-score | -0.002 (-0.009, 0.000) |
| Dietary diversity score → Self-reported questionnaire score → Stimulation → Aggregate development Z-score | 0.000 (0.000, 0.000) |
| Empowerment → Stimulation → Aggregate development Z-score | 0.011 (-0.016, 0.063) |
| Empowerment → Dietary diversity score → Aggregate development Z-score | 0.052 (0.011, 0.126) |
| Empowerment → Self-reported questionnaire score → Aggregate development Z-score | -0.018 (-0.076, 0.004) |
| Empowerment → Self-reported questionnaire score → Stimulation → Aggregate development Z-score | 0.000 (-0.003, 0.000) |
| Empowerment → Dietary diversity score → Self-reported questionnaire score → Aggregate development Z-score | -0.001 (-0.006, 0.000) |
| Empowerment → Dietary diversity score → Self-reported questionnaire score → Stimulation → Aggregate development Z-score | 0.000 (0.000, 0.000) |
| Self-reported questionnaire score → Stimulation → Aggregate development Z-score | 0.000 (0.000, 0.001) |

**Supplemental Table 6** Standardized indirect effects on child development domains through caregiver characteristics^1^

|  | **Gross motor development** | **Fine motor development** | **Language development** | **Social development** |
| --- | --- | --- | --- | --- |
| Dietary diversity score → Self-reported questionnaire score → Domain-specific development Z-score | -0.001 (-0.007, 0.002) | -0.005 (-0.012, -0.001) | 0.001 (-0.003, 0.006) | 0.000 (-0.003, 0.006) |
| Dietary diversity score → Self-reported questionnaire score → Stimulation score → Domain-specific development Z-score | 0.000 (0.000, 0.000) | 0.000 (0.000, 0.000) | 0.000 (0.000, 0.000) | 0.000 (-0.001, 0.000) |
| Empowerment score → Stimulation score → Domain-specific development Z-score | 0.002 (-0.002, 0.009) | -0.001 (-0.006, 0.003) | 0.002 (-0.001, 0.010) | 0.009 (0.002, 0.020) |
| Empowerment score → Dietary diversity score → Domain-specific development Z-score | 0.005 (0.000, 0.015) | 0.001 (-0.003, 0.009) | 0.004 (-0.001, 0.014) | 0.007 (0.001, 0.017) |
| Empowerment score → Self-reported questionnaire score → Domain-specific development Z-score | -0.001 (-0.008, 0.002) | -0.006 (-0.017, -0.001) | 0.001 (-0.004, 0.007) | 0.001 (-0.003, 0.008) |
| Empowerment score → Self-reported questionnaire score → Stimulation score → Domain-specific development Z-score | 0.000 (0.000, 0.000) | 0.000 (0.000, 0.000) | 0.000 (0.000, 0.000) | 0.000 (-0.001, 0.000) |
| Empowerment score → Dietary diversity score → Self-reported questionnaire score → Domain-specific development Z-score | 0.000 (-0.001, 0.000) | 0.000 (-0.001, 0.000) | 0.000 (0.000, 0.000) | 0.000 (0.000, 0.000) |
| Empowerment score → Dietary diversity score → Self-reported questionnaire score → Stimulation score → Domain-specific development Z-score | 0.000 (0.000, 0.000) | 0.000 (0.000, 0.000) | 0.000 (0.000, 0.000) | 0.000 (0.000, 0.000) |
| Self-reported questionnaire score → Stimulation score → Domain-specific development Z-score | 0.000 (-0.001, 0.004) | 0.000 (-0.003, 0.001) | 0.000 (-0.001, 0.005) | 0.001 (-0.005, 0.009) |

^1^ All values are standardized coefficients and bias-corrected bootstrapped 95% confidence intervals in parenthesis.

**Supplemental Table 7** Standardized direct effects of the associations between child development and caregiver factors depicted in the conceptual model, by empowerment indicator^1^

|  | **Empowerment** | **Input into productive decisions** | **Land and other asset ownership** | **Access to and decisions on financial services** | **Control over income use** | **Autonomy in income** | **Group membership** |
| --- | --- | --- | --- | --- | --- | --- | --- |
| Dietary diversity score → Aggregate development Z-score | 0.091  (0.027, 0.143) | 0.091  (0.027, 0.155) | 0.091  (0.026, 0.153) | 0.093  (0.030, 0.155) | 0.093  (0.029, 0.156) | 0.097  (0.033, 0.158) | 0.095  (0.032, 0.159) |
| Dietary diversity score → Self-reported questionnaire score | -0.058  (-0.111, -0.006) | -0.049  (-0.104, 0.005) | -0.044  (-0.099, 0.010) | -0.057  (-0.111, -0.006) | -0.056  (-0.111, -0.002) | -0.063  (-0.116, -0.011) | -0.065  (-0.117, -0.013) |
| Dietary diversity score → Stimulation score | 0.114  (0.054, 0.176) | 0.107  (0.045, 0.173) | 0.113  (0.052, 0.176) | 0.113  (0.054, 0.174) | 0.117  (0.055, 0.180) | 0.118  (0.057, 0.183) | 0.114  (0.054, 0.176) |
| Empowerment score → Aggregate development Z-score | 0.071  (0.007, 0.133) | 0.039  (-0.028, 0.105) | 0.038  (-0.023, 0.097) | 0.030  (-0.034, 0.094) | 0.048  (-0.020, 0.116) | -0.055  (-0.114, 0.011) | 0.000  (-0.061, 0.060) |
| Empowerment score → Dietary diversity score | 0.085  (0.016, 0.145) | 0.135  (0.071, 0.198) | 0.149  (0.074, 0.218) | 0.088  (0.028, 0.142) | 0.071  (0.013, 0.127) | 0.032  (-0.030, 0.091) | 0.071  (0.004, 0.139) |
| Empowerment score → Self-reported questionnaire | -0.068  (-0.137, -0.002) | -0.114  (-0.178, -0.051) | -0.144  (-0.215, -0.069) | -0.071  (-0.135, -0.007) | -0.118  (-0.181, -0.056) | -0.016  (-0.079, 0.048) | -0.016  (-0.048, 0.079) |
| Empowerment score → Stimulation score | 0.074  (0.013, 0.140) | 0.101  (0.044, 0.161) | 0.052  (-0.008, 0.114) | 0.078  (0.020, 0.141) | 0.048  (-0.006, 0.104) | 0.039  (-0.021, 0.096) | 0.080  (0.014, 0.148) |
| Self-reported questionnaire score → Aggregate development Z-score | 0.038  (-0.022, 0.103) | 0.038  (-0.024, 0.102) | 0.038  (-0.024, 0.103) | 0.036  (-0.025, 0.101) | 0.039  (-0.022, 0.105) | 0.033  (-0.028, 0.099) | 0.033  (-0.028, 0.098) |
| Self-reported questionnaire score → Stimulation score | 0.009  (-0.045, 0.068) | 0.014  (-0.040, 0.072) | 0.009  (-0.045, 0.069) | 0.009  (-0.045, 0.069) | 0.009  (-0.045, 0.069) | 0.005  (-0.048, 0.065) | 0.004  (-0.050, 0.063) |
| Stimulation score → Aggregate development Z-score | 0.023  (-0.044, 0.084) | 0.026  (-0.041, 0.087) | 0.028  (-0.038, 0.089) | 0.028  (-0.040, 0.090) | 0.028  (-0.039, 0.089) | 0.032  (-0.035, 0.094) | 0.031  (-0.037, 0.093) |

|  | **Influential group membership** | **Work balance** | **Visiting important locations** | **Respect among household members** | **Attitudes towards intimate partner violence** | **Self-efficacy** |
| --- | --- | --- | --- | --- | --- | --- |
| Dietary diversity score → Aggregate development Z-score | 0.099  (0.034, 0.169) | 0.094  (0.031, 0.157) | 0.096  (0.033, 0.159) | 0.096  (0.033, 0.158) | 0.096  (0.032, 0.160) | 0.090  (0.026, 0.154) |
| Dietary diversity score → Self-reported questionnaire score | -0.258  (-0.470, -0.066) | -0.064  (-0.117, -0.012) | -0.063  (-0.117, -0.011) | -0.063  (-0.117, -0.013) | -0.063  (-0.117, -0.012) | -0.058  (-0.111, -0.005) |
| Dietary diversity score → Stimulation score | 0.174  (0.088, 0.271) | 0.118  (0.056, 0.183) | 0.120  (0.058, 0.182) | 0.120  (0.058, 0.183) | 0.119  (0.057, 0.183) | 0.113  (0.052, 0.174) |
| Empowerment score → Aggregate development Z-score | -0.005  (-0.081, 0.070) | 0.071  (0.012, 0.136) | 0.081  (0.017, 0.142) | 0.110  (0.044, 0.172) | 0.021  (-0.041, 0.083) | 0.085  (0.031, 0.141) |
| Empowerment score → Dietary diversity score | 0.075  (-0.008, 0.160) | 0.018  (-0.035, 0.076) | -0.015  (-0.085, 0.056) | 0.002  (-0.058, 0.059) | -0.025  (-0.082, 0.033) | 0.080  (0.008, 0.146) |
| Empowerment score → Self-reported questionnaire | 0.245  (-0.070, 0.583) | 0.025  (-0.039, 0.088) | 0.052  (-0.014, 0.115) | -0.093  (-0.152, -0.034) | 0.007  (-0.049, 0.065) | -0.069  (-0.139, 0.000) |
| Empowerment score → Stimulation score | 0.132  (0.000, 0.273) | 0.084  (0.036, 0.134) | 0.036  (-0.020, 0.095) | 0.043  (-0.016, 0.097) | -0.004 (-0.051, 0.041) | 0.082  (0.023, 0.136) |
| Self-reported questionnaire score → Aggregate development Z-score | 0.009  (-0.007, 0.027) | 0.032  (-0.029, 0.097) | 0.029  (-0.032, 0.094) | 0.044  (-0.017, 0.108) | 0.033  (-0.028, 0.098) | 0.039  (-0.022, 0.104) |
| Self-reported questionnaire score → Stimulation score | 0.001  (-0.021, 0.025) | 0.003  (-0.053, 0.063) | 0.003  (-0.052, 0.063) | 0.009  (-0.046, 0.069) | 0.005  (-0.049, 0.065) | 0.010  (-0.045, 0.071) |
| Stimulation score → Aggregate development Z-score | 0.020  (-0.024, 0.060) | 0.025  (-0.041, 0.087) | 0.027  (-0.039, 0.087) | 0.025  (-0.041, 0.086) | 0.031  (-0.035, 0.092) | 0.024  (-0.044, 0.084) |

^1^ All values are standardized coefficients and bias-corrected bootstrapped 95% confidence intervals in parenthesis.

**Supplemental Table 8** Standardized indirect effects of the associations between child development and caregiver factors depicted in the conceptual model, by empowerment indicator^1^

|  | **Empowerment** | **Input into productive decisions** | **Land and other asset ownership** | **Access to and decisions on financial services** | **Control over income use** | **Autonomy in income** | **Group membership** |
| --- | --- | --- | --- | --- | --- | --- | --- |
| Dietary diversity score → Self-reported questionnaire score → Aggregate development Z-score | -0.002  (-0.009, 0.001) | -0.002  (-0.008, 0.000) | -0.002  (-0.008, 0.000) | -0.002  (-0.008, 0.001) | -0.002  (-0.009, 0.000) | -0.002  (-0.008, 0.001) | -0.002  (-0.009, 0.001) |
| Dietary diversity score → Self-reported questionnaire score → Stimulation score → Aggregate development Z-score | 0.000  (0.000, 0.000) | 0.000  (0.000, 0.000) | 0.000  (0.000, 0.000) | 0.000  (0.000, 0.000) | 0.000  (0.000, 0.000) | 0.000  (0.000, 0.000) | 0.000  (0.000, 0.000) |
| Empowerment score → Stimulation score → Aggregate development Z-score | 0.002  (-0.002, 0.009) | 0.003  (-0.004, 0.011) | 0.001  (-0.001, 0.009) | 0.002  (-0.002, 0.009) | 0.001  (-0.001, 0.007) | 0.001  (-0.001, 0.008) | 0.002  (-0.002, 0.010) |
| Empowerment score → Dietary diversity score → Aggregate development Z-score | 0.008  (0.002, 0.018) | 0.012  (0.004, 0.025) | 0.014  (0.004, 0.028) | 0.008  (0.002, 0.018) | 0.007  (0.001, 0.016) | 0.003  (-0.002, 0.011) | 0.007  (0.001, 0.019) |
| Empowerment score → Self-reported questionnaire score → Aggregate development Z-score | -0.003  (-0.011, 0.001) | -0.004  (-0.014, 0.002) | -0.006  (-0.018, 0.003) | -0.003  (-0.010, 0.001) | -0.005  (-0.015, 0.002) | -0.001  (-0.006, 0.001) | 0.001  (-0.001, 0.007) |
| Empowerment score → Self-reported questionnaire score → Stimulation score → Aggregate development Z-score | 0.000  (0.000, 0.000) | 0.000  (-0.001, 0.000) | 0.000  (-0.001, 0.000) | 0.000  (0.000, 0.000) | 0.000  (0.000, 0.000) | 0.000  (0.000, 0.000) | 0.000  (0.000, 0.000) |
| Empowerment score → Dietary diversity score → Self-reported questionnaire score → Aggregate development Z-score | 0.000  (0.000, 0.000) | 0.000  (-0.001, 0.000) | 0.000  (-0.001, 0.000) | 0.000  (-0.001, 0.000) | 0.000  (-0.001, 0.000) | 0.000  (-0.001, 0.000) | 0.000  (-0.001, 0.000) |
| Empowerment score → Dietary diversity score → Self-reported questionnaire score → Stimulation score → Aggregate development Z-score | 0.000  (0.000, 0.000) | 0.000  (0.000, 0.000) | 0.000  (0.000, 0.000) | 0.000  (0.000, 0.000) | 0.000  (0.000, 0.000) | 0.000  (0.000, 0.000) | 0.000  (0.000, 0.000) |
| Self-reported questionnaire score → Stimulation score → Aggregate development Z-score | 0.000  (-0.001, 0.005) | 0.000  (-0.001, 0.005) | 0.000  (-0.001, 0.005) | 0.000  (-0.001, 0.005) | 0.000  (-0.001, 0.005) | 0.000  (-0.002, 0.004) | 0.000  (-0.002, 0.004) |

|  | **Influential group membership** | **Work balance** | **Visiting important locations** | **Respect among household members** | **Attitudes towards intimate partner violence** | **Self-efficacy** |
| --- | --- | --- | --- | --- | --- | --- |
| Dietary diversity score → Self-reported questionnaire score → Aggregate development Z-score | -0.002  (-0.009, 0.001) | -0.002  (-0.008, 0.001) | -0.002  (-0.008, 0.001) | -0.003  (-0.009, 0.000) | -0.002  (-0.008, 0.001) | -0.002  (-0.009, 0.000) |
| Dietary diversity score → Self-reported questionnaire score → Stimulation score → Aggregate development Z-score | 0.000  (0.000, 0.000) | 0.000  (0.000, 0.000) | 0.000  (0.000, 0.000) | 0.000  (0.000, 0.000) | 0.000  (0.000, 0.000) | 0.000  (0.000, 0.000) |
| Empowerment score → Stimulation score → Aggregate development Z-score | 0.002  (-0.001, 0.009) | 0.002  (-0.003, 0.009) | 0.001  (-0.001, 0.007) | 0.001  (-0.001, 0.007) | 0.000  (-0.003, 0.001) | 0.002  (-0.003, 0.009) |
| Empowerment score → Dietary diversity score → Aggregate development Z-score | 0.006  (0.000, 0.018) | 0.002  (-0.003, 0.009) | -0.001  (-0.010, 0.005) | 0.000  (-0.006, 0.006) | -0.002  (-0.010, 0.003) | 0.007  (0.001, 0.018) |
| Empowerment score → Self-reported questionnaire score → Aggregate development Z-score | 0.002  (-0.001, 0.010) | 0.001  (-0.001, 0.007) | 0.002  (-0.001, 0.009) | -0.004  (-0.013, 0.001) | 0.000  (-0.001, 0.005) | -0.003  (-0.011, 0.001) |
| Empowerment score → Self-reported questionnaire score → Stimulation score → Aggregate development Z-score | 0.000  (0.000, 0.000) | 0.000  (0.000, 0.000) | 0.000  (0.000, 0.000) | 0.000  (0.000, 0.000) | 0.000  (0.000, 0.000) | 0.000  (-0.001, 0.000) |
| Empowerment score → Dietary diversity score → Self-reported questionnaire score → Aggregate development Z-score | 0.000  (-0.001, 0.000) | 0.000  (0.000, 0.000) | 0.000  (0.000, 0.000) | 0.000  (0.000, 0.000) | 0.000  (0.000, 0.000) | 0.000  (-0.001, 0.000) |
| Empowerment score → Dietary diversity score → Self-reported questionnaire score → Stimulation score → Aggregate development Z-score | 0.000  (0.000, 0.000) | 0.000  (0.000, 0.000) | 0.000  (0.000, 0.000) | 0.000  (0.000, 0.000) | 0.000  (0.000, 0.000) | 0.000  (0.000, 0.000) |
| Self-reported questionnaire score → Stimulation score → Aggregate development Z-score | 0.000  (-0.002, 0.003) | 0.000  (-0.002, 0.004) | 0.000  (-0.002, 0.004) | 0.000  (-0.001, 0.005) | 0.000  (-0.002, 0.004) | 0.000  (-0.001, 0.005) |

^1^ All values are standardized coefficients and bias-corrected bootstrapped 95% confidence intervals in parenthesis.

**Supplemental Table 9** Standardized direct and indirect effects of the associations between child development and caregiver factors depicted in the conceptual model, by subgroup^1^

|  | **Children <6 months of age** | **Children 6-24 months of age** | **Caregivers without education** | **Caregivers with education** | **Married caregivers** | **Unmarried caregivers** |
| --- | --- | --- | --- | --- | --- | --- |
| N | 373 | 648 | 377 | 644 | 864 | 157 |
| *Direct effects* |  |  |  |  |  |  |
| Dietary diversity score → Aggregate development Z-score | 0.133  (0.028, 0.234) | 0.070  (-0.012, 0.152) | 0.064  (-0.037, 0.162) | 0.126  (0.047, 0.201) | 0.093  (0.023, 0.161) | 0.152  (-0.030, 0.311) |
| Dietary diversity score → Self-reported questionnaire | -0.009  (-0.111, 0.096) | -0.071  (-0.139, -0.006) | -0.091  (-0.175, -0.006) | -0.008  (-0.081, 0.071) | -0.031  (-0.092, 0.028) | -0.137  (-0.266, -0.002) |
| Dietary diversity score → Stimulation score | 0.207  (0.088, 0.309) | 0.104  (0.031, 0.179) | 0.106  (-0.003, 0.215) | 0.113  (0.037, 0.190) | 0.133  (0.068, 0.200) | -0.026  (-0.197, 0.147) |
| Empowerment score → Aggregate development Z-score | 0.077  (-0.052, 0.205) | 0.085  (0.012, 0.155) | 0.088  (-0.022, 0.191) | 0.076  (-0.003, 0.155) | 0.100  (0.024, 0.170) | -0.008  (-0.150, 0.140) |
| Empowerment score → Dietary diversity score | 0.154  (0.035, 0.255) | 0.075  (0.005, 0.144) | 0.059  (-0.050, 0.166) | 0.130  (0.051, 0.208) | 0.087  (0.016, 0.153) | 0.107  (-0.061, 0.267) |
| Empowerment score → Self-reported questionnaire | -0.117  (-0.234, -0.002) | -0.024  (-0.104, 0.050) | -0.004  (-0.094, 0.090) | -0.091  (-0.177, -0.007) | -0.055  (-0.133, 0.019) | -0.091  (-0.226, 0.064) |
| Empowerment score → Stimulation score | -0.033  (-0.152, 0.091) | 0.135  (0.062, 0.214) | 0.101  (0.013, 0.193) | 0.041  (-0.032, 0.117) | 0.036  (-0.031, 0.101) | 0.221  (0.049, 0.382) |
| Self-reported questionnaire → Aggregate development Z-score | 0.039  (-0.067, 0.157) | 0.019  (-0.050, 0.090) | 0.054  (-0.044, 0.147) | 0.026  (-0.049, 0.109) | 0.057  (-0.008, 0.128) | -0.046  (-0.171, 0.082) |
| Self-reported questionnaire → Stimulation score | -0.042  (-0.144, 0.063) | 0.035  (-0.048, 0.121) | 0.056  (-0.032, 0.155) | -0.047  (-0.123, 0.032) | 0.001  (-0.066, 0.071) | -0.046  (-0.197, 0.094) |
| Stimulation score → Aggregate development Z-score | -0.091  (-0.192, 0.016) | 0.212  (0.137, 0.286) | 0.048  (-0.051, 0.143) | 0.016  (-0.064, 0.098) | 0.041  (-0.026, 0.108) | 0.016  (-0.171, 0.184) |
|  |  |  |  |  |  |  |
| *Indirect effects* |  |  |  |  |  |  |
| Dietary diversity score → Self-reported questionnaire → Aggregate development Z-score | 0.000  (-0.012, 0.004) | -0.001  (-0.009, 0.003) | -0.005  (-0.022, 0.002) | 0.000  (-0.005, 0.002) | -0.002  (-0.009, 0.001) | 0.006  (-0.008, 0.035) |
| Dietary diversity score → Self-reported questionnaire → Stimulation score → Aggregate development Z-score | 0.000  (-0.001, 0.000) | -0.001  (-0.003, 0.000) | 0.000  (-0.002, 0.000) | 0.000  (0.000, 0.000) | 0.000  (0.000, 0.000) | 0.000  (-0.001, 0.004) |
| Empowerment score → Stimulation score → Aggregate development Z-score | 0.003  (-0.006, 0.023) | 0.029  (0.012, 0.051) | 0.005  (-0.003, 0.021) | 0.001  (-0.002, 0.009) | 0.001  (-0.001, 0.009) | 0.004  (-0.044, 0.046) |
| Empowerment score → Dietary diversity score → Aggregate development Z-score | 0.020  (0.003, 0.052) | 0.005  (0.000, 0.018) | 0.004  (-0.002, 0.023) | 0.016  (0.005, 0.035) | 0.008  (0.001, 0.021) | 0.016  (-0.005, 0.078) |
| Empowerment score → Self-reported questionnaire score → Aggregate development Z-score | -0.005  (-0.025, 0.006) | 0.000  (-0.007, 0.001) | 0.000  (-0.009, 0.006) | -0.002  (-0.013, 0.003) | -0.003  (-0.013, 0.000) | 0.004  (-0.006, 0.033) |
| Empowerment score → Self-reported questionnaire score → Stimulation score → Aggregate development Z-score | 0.000  (-0.004, 0.000) | 0.000  (-0.002, 0.000) | 0.000  (-0.001, 0.000) | 0.000  (0.000, 0.001) | 0.000  (0.000, 0.000) | 0.000  (-0.001, 0.003) |
| Empowerment score → Dietary diversity score → Self-reported questionnaire score → Aggregate development Z-score | 0.000  (-0.002, 0.001) | 0.000  (-0.001, 0.000) | 0.000  (-0.003, 0.000) | 0.000  (-0.001, 0.000) | 0.000  (-0.001, 0.000) | 0.001  (-0.001, 0.007) |
| Empowerment score → Dietary diversity score → Self-reported questionnaire score → Stimulation score → Aggregate development Z-score | 0.000  (0.000, 0.000) | 0.000  (0.000, 0.000) | 0.000  (0.000, 0.000) | 0.000  (0.000, 0.000) | 0.000  (0.000, 0.000) | 0.000  (0.000, 0.001) |
| Self-reported questionnaire score → Stimulation score → Aggregate development Z-score | 0.004  (-0.003, 0.023) | 0.007  (-0.009, 0.029) | 0.003  (-0.002, 0.016) | -0.001  (-0.010, 0.003) | 0.000  (-0.004, 0.004) | -0.001  (-0.024, 0.010) |

^1^ All values are standardized coefficients and bias-corrected bootstrapped 95% confidence intervals in parenthesis.

**Enrolled in baseline survey**

n=2,686

**Pregnant at baseline**

n=1,305

**Have a child <2 years of age at baseline**

n=1,381

**Missing data on outcomes and factors of interest**

n=360

**Final analytic sample**

n=1,021

**Supplemental Figure 1** Study flow diagram.

Number of stimulation activities provided by the mother

Empowerment score

Self-reported questionnaire score

Dietary diversity score

0.094 (0.029, 0.163)

Aggregate development Z-score

0.481 (0.048, 0.901)

0.004 (-0.019, 0.028)

0.015 (-0.029, 0.054)

0.010 (-0.006, 0.028)

0.772 (0.133, 1.469)

0.055 (0.103, 0.946)

-1.711 (-3.455, -0.052)

-0.224 (-0.440, -0.024)

0.114 (0.054, 0.176)

**Supplemental Figure 2** Unstandardized direct effects and bias-corrected bootstrapped 95% confidence intervals. Grey paths represent estimates not significant at the 5% level. Estimates controlled for child age and sex, caregiver age, education, and marital status, household size and expenditures, and district. The direct paths to MDAT, which is age-standardized, did not control for child age. The direct paths to MDAT and stimulation practices also controlled for length-for-age Z-score. Model fit statistics: CFI=1.000, RMSEA=0.000, and SRMR=0.006.

Number of stimulation activities provided by the mother

Empowerment score

Self-reported questionnaire score

Dietary diversity score

0.064 (0.001, 0.124)

Gross motor development Z-score

0.043 (-0.018, 0.101)

0.009 (-0.045, 0.068)

0.022 (-0.039, 0.086)

0.017 (-0.045, 0.083)

0.074 (0.013, 0.140)

0.085 (0.016, 0.145)

-0.068 (-0.137, -0.002)

-0.058 (-0.111, -0.006)

0.114 (0.054, 0.176)

**Supplemental Figure 3** Standardized direct effects and bias-corrected bootstrapped 95% confidence intervals. Grey paths represent estimates not significant at the 5% level. Estimates controlled for child age and sex, caregiver age, education, and marital status, household size and expenditures, and district. The direct paths to MDAT, which is age-standardized, did not control for child age. The direct paths to MDAT and stimulation practices also controlled for length-for-age Z-score. Model fit statistics: CFI=1.000, RMSEA=0.000, and SRMR=0.006.

Number of stimulation activities provided by the mother

Empowerment score

Self-reported questionnaire score

Dietary diversity score

0.016 (-0.044, 0.075)

Fine motor development Z-score

0.033 (-0.037, 0.101)

0.009 (-0.045, 0.068)

-0.013 (-0.071, 0.046)

0.084 (0.024, 0.151)

0.074 (0.013, 0.140)

0.085 (0.016, 0.145)

-0.068 (-0.137, -0.002)

-0.058 (-0.111, -0.006)

0.114 (0.054, 0.176)

**Supplemental Figure 4** Standardized direct effects and bias-corrected bootstrapped 95% confidence intervals. Grey paths represent estimates not significant at the 5% level. Estimates controlled for child age and sex, caregiver age, education, and marital status, household size and expenditures, and district. The direct paths to MDAT, which is age-standardized, did not control for child age. The direct paths to MDAT and stimulation practices also controlled for length-for-age Z-score. Model fit statistics: CFI=0.975, RMSEA=0.038, and SRMR=0.014.

Number of stimulation activities provided by the mother

Empowerment score

Self-reported questionnaire score

Dietary diversity score

0.050 (-0.021, 0.125)

Language development Z-score

0.086 (0.017, 0.149)

0.009 (-0.045, 0.068)

0.032 (-0.029, 0.091)

-0.009 (-0.072, 0.055)

0.074 (0.013, 0.140)

0.085 (0.016, 0.145)

-0.068 (-0.137, -0.002)

-0.058 (-0.111, -0.006)

0.114 (0.054, 0.176)

**Supplemental Figure 5** Standardized direct effects and bias-corrected bootstrapped 95% confidence intervals. Grey paths represent estimates not significant at the 5% level. Estimates controlled for child age and sex, caregiver age, education, and marital status, household size and expenditures, and district. The direct paths to MDAT, which is age-standardized, did not control for child age. The direct paths to MDAT and stimulation practices also controlled for length-for-age Z-score. Model fit statistics: CFI=1.000, RMSEA=0.000, and SRMR=0.006.

Number of stimulation activities provided by the mother

Empowerment score

Self-reported questionnaire score

Dietary diversity score

0.082 (0.019, 0.143)

Social development Z-score

0.074 (0.010, 0.135)

0.009 (-0.045, 0.068)

0.016 (0.048, 0.178)

-0.008 (-0.075, 0.057)

0.074 (0.013, 0.140)

0.085 (0.016, 0.145)

-0.068 (-0.137, -0.002)

-0.058 (-0.111, -0.006)

0.114 (0.054, 0.176)

**Supplemental Figure 6** Standardized direct effects and bias-corrected bootstrapped 95% confidence intervals. Grey paths represent estimates not significant at the 5% level. Estimates controlled for child age and sex, caregiver age, education, and marital status, household size and expenditures, and district. The direct paths to MDAT, which is age-standardized, did not control for child age. The direct paths to MDAT and stimulation practices also controlled for length-for-age Z-score. Model fit statistics: CFI=0.939, RMSEA=0.062, and SRMR=0.019.
